# Diabetes Screening in the Emergency Department: Development of a Predictive Model for Elevated Hemoglobin A1c

**DOI:** 10.1155/jdr/8830658

**Published:** 2025-03-12

**Authors:** Mary H. Smart, Janet Y. Lin, Brian T. Layden, Yuval Eisenberg, A. Simon Pickard, Lisa K. Sharp, Kirstie K. Danielson, Angela Kong

**Affiliations:** ^1^Department of Pharmacy Systems, Outcomes and Policy, College of Pharmacy, The University of Illinois Chicago, Chicago, Illinois, USA; ^2^Department of Emergency Medicine, College of Medicine, The University of Illinois Chicago, Chicago, Illinois, USA; ^3^Division of Endocrinology, Diabetes and Metabolism, Department of Medicine, The University of Illinois Chicago, Chicago, Illinois, USA; ^4^Jesse Brown Veterans Affairs Medical Center, Chicago, Illinois, USA; ^5^Department of Biobehavioral Nursing Science, College of Nursing, The University of Illinois Chicago, Chicago, Illinois, USA

## Abstract

**Aims:** We developed a prediction model for elevated hemoglobin A1c (HbA1c) among patients presenting to the emergency department (ED) at risk for diabetes to identify important factors that may influence follow-up patient care.

**Methods:** Retrospective electronic health records data among patients screened for diabetes at the ED in May 2021 was used. The primary outcome was elevated HbA1c (≥ 5.7%). The data was divided into a derivation set (80%) and a test set (20%) stratified by elevated HbA1c. In the derivation set, we estimated the optimal significance level for backward elimination using a 10-fold cross-validation method. A final model was derived using the entire derivation set and validated on the test set. Performance statistics included C-statistic, sensitivity, specificity, predictive values, Hosmer–Lemeshow test, and Brier score.

**Results:** There were 590 ED patients screened for diabetes in May 2021. The final model included nine variables: age, race/ethnicity, insurance, chief complaints of back pain and fever/chills, and a past medical history of obesity, hyperlipidemia, chronic obstructive pulmonary disease, and substance misuse. Adequate model discrimination (C−statistic = 0.75; sensitivity, specificity, and predictive values > 0.70), no evidence of model ill fit (Hosmer–Lemeshow test = 0.29), and moderate Brier score (0.21) suggest acceptable model performance.

**Conclusion:** In addition to age, obesity, and hyperlipidemia, a history of substance misuse was identified as an important predictor of elevated HbA1c levels among patients screened for diabetes in the ED. Our findings suggest that substance misuse may be an important factor to consider when facilitating follow-up care for patients identified with prediabetes or diabetes in the ED and warrants further investigation. Future research efforts should also include external validation in larger samples of ED patients.

## 1. Introduction

There are over 37 million US adults with diabetes, and of those, 20% are unaware that they have diabetes [1]. The United States Preventive Task Force recommends routine screening of adults at risk for diabetes, which has typically been conducted in the primary care setting [2]. However, certain populations, such as racially and ethnically minoritized populations, may face barriers to primary care utilization and, as a result, are less likely to be screened for diabetes [3]. Diabetes screening in the acute care setting may act as a safety net for these populations, who tend to use emergency care services more frequently [3–5]. However, connecting patients screened positive for either diabetes or prediabetes among previous efforts to implement diabetes screening within the emergency department (ED) to follow-up care has been poor [6, 7]. Understanding the characteristics of patients identified with newly diagnosed diabetes or prediabetes in the ED may be important to inform strategies linking these patients to services within primary care.

The ED stands apart from other healthcare settings because it provides accessible and timely care for an array of serious and acute medical situations, regardless of an individual's ability to pay [8]. This contrasts with primary care, which often requires scheduling appointments ahead of time, is unavailable 24/7, and may only accept certain types of insurance [4]. Consequently, the ED setting often acts as a safety net for diverse patient populations [5]. For example, the ED population tends to have a higher proportion of individuals with serious illness or injury who are seeking immediate care compared to the general population [4]. Additionally, racially and ethnically minoritized populations are more likely to use emergency services as a safety-net facility than other health services due to barriers to primary care access, needing timely access to care, and mistrust of the medical community [3, 9]. Furthermore, since the ED serves patients regardless of their insurance status, it is more likely to see uninsured and underinsured populations than other healthcare settings [10]. Ultimately, the unique function of the ED leads to differences between those who frequent the ED and the general patient population. As a result, the ED setting, with its distinct patient population, makes it challenging for one-size-fits-all prediction models to accurately capture the intricacies of the acute care setting [11]. Therefore, if a prediction model is developed primarily on the general patient population, its applicability to specific populations, like those seen in the ED, may be limited [12].

Clinical prediction models are useful tools to help identify patients at an increased risk for a medical condition and aid healthcare professionals in making informed decisions about patient care [13, 14]. Several diabetes prediction models and risk assessment tools have been developed to estimate individuals at risk of developing diabetes [15–17]. However, most of these models have been developed and validated in populations not representative of those seen in the acute care setting [18–20]. As a result, there may be factors specific to patients presenting to the ED that are not represented in current risk prediction tools [12, 19]. These factors may be important to consider in developing and implementing effective strategies for linking ED patients to prevention and treatment services beyond the ED setting. Therefore, we aimed to develop a prediction model of elevated hemoglobin A1c (HbA1c) among those screened for diabetes within the ED using demographic and clinical characteristics measured during the ED visit. Our goal was to identify predictors of elevated HbA1c within the acute care setting to better understand the patient population served by the ED-based diabetes screening program.

## 2. Materials and Methods

### 2.1. Background

In 2020, the University of Illinois Hospital and Health Sciences System, a large urban academic medical center in Chicago serving a predominantly racially and ethnically minoritized population and Medicaid recipients, implemented a routine, electronic medical record (EMR)-based diabetes screening program within the ED. The goal of the program was to identify patients with undiagnosed prediabetes or diabetes and facilitate linkage to primary care. An HbA1c test using high-performance liquid chromatography was added to an existing lab order for eligible patients identified using a best practice alert embedded in the EMR informed by the American Diabetes Association's guidelines. HbA1c values ≥ 5.7% were considered elevated. Patients with a previous diagnosis of diabetes were ineligible for HbA1c testing and excluded from the cohort. A further description of the program is provided elsewhere [21].

### 2.2. Study Design and Source of Data

We conducted a cross-sectional study using data extracted from the EMR. Data was collected using automatically generated EMR reports and manual data extraction. Manual chart extraction used a double-entry method, and personnel entering data met regularly to resolve discrepancies. The study followed the TRIPOD (transparent reporting of a multivariable prediction model for individual prognosis or diagnosis) guidance for prediction model development and validation [22]. The University of Illinois Chicago's Institutional Review Board reviewed and approved this study (Protocol: 2021-1048).

### 2.3. Sample Population

The study included all adult patients (≥ 18 years) screened for diabetes in the ED in May 2021. Patients were screened based on American Diabetes Association screening guidelines using an EMR-based algorithm [23]. Eligible patients for screening were as follows: (1) those ≥ 45 years of age or (2) ≥18 years with BMI ≥ 25 kg/m^2^. Any patients with a previous diagnosis of diabetes or who had an HbA1c test recorded in their hospital medical record within 3 years of their ED visit were ineligible for screening.

### 2.4. Outcome Measure

The prediction outcome of interest was an elevated (i.e., ≥ 5.7%) HbA1c value, indicative of possible undiagnosed prediabetes or diabetes among those who were screened for diabetes in the ED. The outcome was dichotomized (i.e., elevated vs. normal (< 5.7%) HbA1c value).

### 2.5. Candidate Variables

Among the baseline variables collected, candidate variables were selected based on clinical expertise and previous literature on factors associated with prediabetes and diabetes [24]. We assessed for multicollinearity between variables using a correlation matrix and variance inflation factor (> 5) [25]. Investigators (M.H.S. and A.K.) met to decide which variables to retain among those that exhibited correlations. Bivariate analysis was conducted for each candidate predictor and the outcome of interest using a chi-square test and *t*-test for categorical and continuous variables, respectively. Variables that exhibited a significant association with the outcome at a *p* value of < 0.20 were retained for model evaluation. Of the candidate variables evaluated, 17 candidate predictor variables were retained for multivariable regression model development and validation.

The final candidate variables included patient age (years), sex (male, female), and race/ethnicity (non-Hispanic White; non-Hispanic Black or African American; non-Hispanic Asian; Hispanic, Latino(a), or Spanish origin; or other/unknown). Insurance types included commercial/private, Medicaid, Medicare, and other/unknown/uninsured. Past medical history was defined as any current or past major illnesses or comorbidities listed in the patient's medical record or mentioned in the doctors' notes during the ED visit. The patient's past social history was not evaluated. Patients presenting with a BMI ≥ 30 kg/m^2^ were identified as obese. Substance misuse was defined as any (poly)substance disorder, misuse, or abuse of any of the following: alcohol, opioids, nicotine/tobacco, and cannabis noted within the patient's past medical history or during the current ED visit. Cardiovascular disease included medical history of myocardial infarction, venous thromboembolism, and atherothrombosis. A chief complaint is defined as the main reason for ED visits. The candidate predictors were assessed for missingness. Candidate predictors displayed no more than 5% missingness and none for the outcome.

### 2.6. Statistical Analysis

Data cleaning and analysis were conducted using SAS 9.4 software (SAS Institute, Cary, North Carolina, United States). The cohort was split into a derivation set (80%) and a test set (20%), stratified by HbA1c status (i.e., elevated vs. normal) to ensure similar outcome proportions across the two datasets. Descriptive statistics were performed on the candidate predictors using frequencies and proportions for categorical and mean with standard deviations for continuous variables on the total, derivation, and test sets.

Multivariable logistic regression using backward elimination was based on the Akaike information criteria to account for a small sample size [26]. The optimal significance value for variable retention was determined by conducting a 10-fold cross-validation of logistic regression models using backward elimination based on significance levels of 0.01, 0.05, 0.157, and 0.25 [27]. These were compared to a model including all candidate variables. Our aim in using a 10-fold cross-validation technique was to identify the most ideal significance level, avoiding a *p* value too small that would exclude important predictors or too high a value that resulted in including unnecessary erroneous predictors [26]. Additionally, the frequency by which the parameters were selected across the different model strategies was compiled. Performance measures for each model were compared to determine the optimal *p*-value for the final model.

A final model was derived using the entire derivation set and then internally validated on the test set. The C-statistic, sensitivity, and specificity were used to determine model discrimination, which is how well the model can discriminate between those with elevated versus normal HbA1c levels [28]. The Hosmer–Lemeshow test was used to assess model fit and calibration, which is the level of agreement between observed probability and predicted probability of the outcome [26, 29]. Scaled Brier score, a measure assessing both discrimination and calibration, was used to represent overall prediction performance (with performance values depending on outcome prevalence) [29].

## 3. Results

### 3.1. Participant Characteristics

There were 3290 ED admissions in May 2021, with 590 (18%) adults screened for diabetes that were included in this study. Demographic and clinical characteristics of the cohort using candidate variables are reported in Table 1. In general, those with elevated HbA1c levels (i.e., ≥ 5.7%) were older (57 vs. 48, *p* < 0.001). There were also differences in racial/ethnic backgrounds across the two groups, with more non-Hispanic Blacks or African Americans and non-Hispanic Asians within the elevated HbA1c group (*p* = 0.04). Compared to those with normal HbA1c levels, those with elevated HbA1c were more likely to have comorbidities or a past medical history of hypertension, obesity, hyperlipidemia, cardiovascular disorder, arthritis, chronic obstructive pulmonary disease (COPD), cancer, and substance misuse. Finally, those with elevated HbA1c levels were more likely to present to the ED with a chief complaint of back pain and a chief complaint of fever and/or chills than those with normal HbA1c levels.

The derivation set contained 457 observations with 208 (45.5%) abnormal HbA1c levels, and the test set contained 113 observations with 51 (45.1%) elevated HbA1c levels. A comparison of the derivation and test set across the candidate variables can be seen in Appendix Table S1. There was no statistically significant difference between the derivation and test cohort except for the race/ethnicity category (*p* < 0.01), which was driven mainly by the non-Hispanic Asian category.

### 3.2. Model Derivation

There were 50 models generated using the 10-fold cross-validation. All models displayed no evidence of poor fit. While there was no statistical difference between the modeling strategies (i.e., models of differing *p* values and all predictors) in C-statistic (*p* = 0.65) or Brier score (*p* = 0.71), the modeling strategy with the highest average C-statistic (0.74) and lowest average Brier score (0.21) was *p* value = 0.157. Box plots of the average C-statistics and Brier score, along with means and standard deviations, can be seen in Appendix Figures S1 and S2. The variables that were selected at least 75% across all 50 models were as follows: a history of obesity, substance misuse, and hyperlipidemia;age; insurance status; and a chief complaint of back pain (Appendix Table S2).

Our final model was developed on the entire derivation dataset using a multivariable logistic regression model with backward elimination at a significance level of 0.157.  Table 2 shows the point estimates for the final model with standard errors and 95% confidence intervals. The final model included nine variables, which were age, race/ethnicity, insurance, and a past medical history of obesity, hyperlipidemia, COPD, and substance misuse, along with chief complaints of back pain and fever/chills.

### 3.3. Internal Validation

The performance of the final model on the test cohort can be seen in Table 3. The prediction model demonstrated adequate ability to discriminate between normal versus elevated HbA1c among the ED diabetes screened population, as indicated by a C-statistic of 0.75 and sensitivity and specificity > 0.70 [28]. The negative predictive value was 0.73, while the positive predictive value was 0.67, which suggests that the model has better discriminative ability in detecting the absence of the outcome given a normal HbA1c result [30].

A visual comparison of the area under the receiver operating characteristic curve (AUC) for the final model on the derivation (0.77) versus the test cohort (0.75) can be seen in Appendix Figure S3. The Hosmer–Lemeshow test was not statistically significant (*p* = 0.29), indicating adequate calibration [28]. The Brier score was 0.21, which is moderate given the outcome proportion was almost half the population and may suggest a need for to update the algorithm during future validation efforts [28].

## 4. Discussion

We developed a prediction model for elevated HbA1c among ED patients determined to be at risk for diabetes. Our goal was to identify important predictors of elevated HbA1c specific to the acute care setting that may not be reflected in current diabetes risk tests typically administered in the primary care setting [2]. To our knowledge, this is the first prediction model of elevated HbA1c among those screened for diabetes in an acute care setting and can have important implications for diabetes screening initiatives within the ED. Our findings highlighted that in addition to age, obesity, and hyperlipidemia, a history of substance misuse is an important predictor of elevated HbA1c in the acute care setting. These findings may suggest an important role for substance misuse, though further research is needed to elucidate its relationship with elevated HbA1c results (i.e., casual versus noncasual relationship).

Four variables were selected 90% of the time across the k-fold models: age, and a history of substance misuse, obesity, and hyperlipidemia. The association of age, obesity, and hyperlipidemia with diabetes and prediabetes is well established [31, 32]. Interestingly, our model identified substance misuse as a significant predictor of elevated HbA1c among at-risk patients in the ED. This suggests that a history of substance misuse may be an important factor to consider when facilitating postscreening care, such as diabetes prevention or management in primary care, for ED patients with a potentially new diagnosis of prediabetes and diabetes.

The prevalence of individuals with a history of substance misuse presenting to the ED likely explains the presence and importance of substance misuse in our predictive model. In 2021, substance misuse, including alcohol and illicit drug misuse, abuse, and dependency, is estimated to be present in over 11% of ED visits in the United States [33] Our study aligns with national reports, with a prevalence of 9% substance misuse in our study cohort. Patients with a history of substance misuse are more likely to frequent emergency services rather than engage with primary care [16, 34]. Furthermore, attempts to link patients to primary care from the ED with a history of substance misuse have been met with challenges due to reported barriers such as stigma and patient-experienced healthcare costs [35–37]. Therefore, considering the characteristics and needs of patients with a history of substance misuse may be essential to successfully linking ED patients identified with elevated HbA1c for follow-up treatment and prevention services within routine care and warrants further examination.

Additionally, literature has reported poor glycemic control among patients with a history of substance use disorder, a clinical diagnosis for patients with chronic misuse of substances [38, 39]. Alcohol and recreational drug use are associated with the incidence of Type 2 diabetes [40, 41]. Patients with a history of substance misuse may have poor prediabetes and diabetes-related outcomes due to the impact of social determinants of health [42]. Social determinants, such as food and housing insecurity, lack of neighborhood safety, and lack of insurance, make diabetes prevention and management challenging for these patients since treatment regimens often include intense lifestyle modification (i.e., healthy eating and physical activity) and/or prescription medication [38, 41, 43]. Patients unable to engage in lifestyle modification, access, and adhere to treatment regimens due to their circumstances and environment that require extraordinary efforts to overcome barriers to being healthy will be less likely to manage their prediabetes or diabetes [43, 44]. On the other hand, engagement with treatment of substance misuse is associated with improved HbA1c control [38, 39]. Our findings suggest the successful linkage and delivery of diabetes treatment and prevention services in primary care may need to address substance misuse and interventions sensitive to social determinants of health.

Our prediction model differs from other prominent diabetes and prediabetes prediction models in several ways. First, most reported diabetes prediction models, such as the American Diabetes Association's diabetes and prediabetes risk calculators, have been developed and validated in the general population, which is typically utilized to inform routine care [2, 15, 18–20]. To identify characteristics specific to patients seen in acute care that may influence postscreening referral to primary care, the source population for our model was a subset of patients already determined to be at risk for diabetes who presented to the ED. Additionally, we did not include a family history of diabetes or prediabetes as a candidate predictor since it is often poorly documented within patient medical charts, particularly among certain ethnically minoritized populations [45]. Finally, previous diabetes risk assessment tools have identified hypertension to be a strong risk factor for diabetes and prediabetes [15, 18]. However, the covariate for a history of hypertension was not included in our final model. A studentized *t*-test showed that age was strongly associated (*p* < 0.01) with hypertension and other comorbidities like obesity, hyperlipidemia, and cardiovascular disorder despite not meeting the threshold for elimination for multicollinearity during model development.

Our study has some notable limitations. First, our sample size is relatively small, which may lead to unstable parameter estimates and overfitting [46]. However, we utilized the Akaike information criterion to account for the small sample size [26]. Future studies in larger external ED populations will be necessary to validate the model further and to possibly update the algorithm. We did not utilize data imputation given that our outcome and candidate predictors were nearly complete and had a relatively small sample size, which may have resulted in inefficiencies and potential bias predictors [26]. Also, variables associated with past medical history, including substance misuse, may have included past but not active medical conditions.

Additionally, our study cohort is comprised of those who were screened for diabetes within the ED. As expected, the baseline characteristics of those who were screened and not screened were slightly different since the screening algorithm used to identify at-risk patients included parameters set by the American Diabetes Association screening guidelines. For example, those who were screened versus not screened were older (51 vs. 46, *p* < 0.01) and more likely male (50% vs. 36%, *p* < 0.01). Therefore, our conclusions can only be generalized to those deemed at-risk for diabetes within the ED and not to the entire ED population.

Importantly, since the goal of the study was to develop a predictive model and optimize the predictive power of a group of variables, it would be misleading to interpret the individual beta coefficients [47, 48]. Therefore, we are unable to make causal inferences of, for example, a history of substance misuse on having an elevated abnormal HbA1c level. However, our model does point to the possible importance of a history of substance misuse in being at risk for diabetes among patients presenting to the ED that warrants further exploration.

Furthermore, our study sample was collected during the COVID-19 pandemic, which may affect the generalizability of our findings. For example, a chief complaint of fever and chills was an important predictor in our model, which suggests the influence of the COVID-19 pandemic on our cohort utilized in building the model. Patient hospitalizations and ED admissions varied considerably before, during, and after the pandemic attributed to the COVID-19 lockdowns and restrictions [49, 50]. Therefore, the types of patients presenting to the ED during the COVID-19 lockdown may not reflect those seen beyond the pandemic period. However, we believe the implications of our predictive model are relevant to the ED population irrespective of the COVID-19 pandemic since the prevalence of substance misuse has been pervasive before and through the pandemic [51, 52]. Future studies should evaluate the model's validity in populations unaffected by a major health event like the COVID-19 pandemic.

In conclusion, our model provides insight into important ED patient characteristics predictive of elevated HbA1c that are not captured in existing diabetes and prediabetes prediction models. In addition to age, obesity, and hyperlipidemia, a history of substance misuse was identified as an important predictor of elevated HbA1c among patients screened for diabetes in the ED. Considering potential substance misuse among ED patients may be critical for successful linkage to primary care and management of hyperglycemia beyond the acute care setting. Our work informs ongoing development and strategies of existing diabetes screening initiatives within and beyond the acute care setting. Future research efforts include external validation in larger ED populations, including broader time periods, rural settings, and regions beyond the Chicago metropolitan area.

## Figures and Tables

**Table 1:** Sample characteristics of cohort as described by candidate predictor variables and associations with elevated HbA1c (*n* = 570).

	**HbA1c test**				
	**Normal (< 5.7%)** **n** = 331		**Elevated (≥ 5.7%)** **n** = 259		**p** ** value**
Age, mean (SD)	47.9	(17.7)	56.6	(14.9)	< 0.001
Sex, *n* (%)					
Female	162	(52.1)	124	(47.9)	0.32
Male	149	(47.9)	135	(52.1)	
Race/ethnicity, *n* (%)					
Non-Hispanic, White	59	(19.0)	34	(13.1)	0.04
Non-Hispanic, Black or African American	125	(40.2)	128	(49.4)	
Non-Hispanic, Asian	12	(3.9)	18	(6.9)	
Hispanic, Latino/a, or Spanish origin	97	(31.2)	69	(26.6)	
Other/unknown	18	(5.8)	10	(3.9)	
Insurance, *n* (%)					
Commercial/private	91	(29.3)	67	(25.9)	0.34
Medicaid	120	(38.6)	93	(35.9)	
Medicare	76	(24.4)	69	(26.6)	
Other/unknown/uninsured	24	(7.7)	30	(11.6)	
Past medical history, *n* (%)					
Hypertension	98	(31.5)	124	(47.9)	< 0.001
Obesity	117	(37.6)	121	(46.7)	0.03
Hyperlipidemia	18	(5.8)	46	(17.8)	< 0.001
Cardiovascular disorder	34	(10.9)	47	(18.1)	0.01
Arthritis	14	(4.5)	26	(10.0)	0.01
COPD	8	(2.6)	28	(10.8)	< 0.001
Cancer	19	(6.1)	33	(12.7)	0.01
Substance misuse	18	(5.8)	35	(13.5)	0.02
Heart failure	12	(3.9)	16	(6.2)	0.20
Atrial fibrillation	12	(3.9)	16	(6.2)	0.20
Chief complaint included, *n* (%)					
Back pain	4	(1.3)	13	(5.0)	0.01
Fever/chills	5	(1.6)	17	(6.6)	< 0.001
Nausea/vomiting	19	(6.1)	26	(10.0)	0.08

Abbreviations: COPD, chronic obstructive pulmonary disease; HbA1c, glycated hemoglobin.

**Table 2:** Parameter estimates for final model.

**Parameter**	**Estimate**	**Standard error**	**Pr** > |**t**|	**95% confidence limits**	
Intercept	−3.724	0.566	< 0.001	−4.833	−2.615
Age	0.045	0.009	< 0.001	0.028	0.062
Race/ethnicity: Hispanic, Latino/a, or Spanish origin	0.172	0.350	0.623	−0.514	0.858
Race/ethnicity: non-Hispanic, Asian	1.313	0.509	0.010	0.316	2.311
Race/ethnicity: non-Hispanic, Black or African American	0.607	0.318	0.056	−0.016	1.229
Race/ethnicity: other/unknown	0.114	0.528	0.829	−0.920	1.148
Insurance: Medicaid	0.187	0.280	0.504	−0.362	0.736
Insurance: Medicare	−0.754	0.348	0.030	−1.435	−0.073
Insurance: other/unknown/uninsured	0.916	0.429	0.033	0.075	1.757
Obesity	0.939	0.240	< 0.001	0.467	1.410
Hyperlipidemia	1.195	0.386	0.002	0.438	1.952
Chronic obstructive pulmonary disease	1.138	0.522	0.029	0.116	2.161
Substance misuse	1.422	0.426	0.001	0.588	2.256
Chief complaint: back pain	1.674	0.666	0.012	0.370	2.978
Chief complaint: fever/chills	1.507	0.635	0.018	0.262	2.752

**Table 3:** Performance fit statistic for final model.

**Performance fit statistic**	
**Type**	**Final model**
C-statistic	0.747
Hosmer–Lemeshow test	0.291
Brier score	0.205
True negative fraction (specificity)	0.710
True positive fraction (sensitivity)	0.700
Positive predictive value	0.673
Negative predictive value	0.735

## Data Availability

The data that supports the findings of this study may be available upon request from the corresponding author. The data is not publicly available due to privacy or ethical restrictions.

## Supporting Information

Supporting Information 1 Appendix Table S1: a comparison of the derivation and test set across the candidate variables.

Supporting Information 2 Appendix Table S2: frequency of variables selected across 40 models (excluding models with all variables).

Supporting Information 3 Appendix Figure S1: box plot of the average C-statistic across the 10-folds for each model with varying *p* values.

Supporting Information 4 Appendix Figure S2: box plot for the average Brier score across 10-folds for each model with varying *p* values.

Supporting Information 5 Appendix Figure S3: the receiver operating characteristic curve (AUC) for the final model on the derivation and test cohort.
