# Supplementary material for: Diabetes Screening in the Emergency Department: Development of a Predictive Model for Elevated Hemoglobin A1c
Source: J Diabetes Res. 2025 Mar 12;2025:8830658. doi: 10.1155/jdr/8830658 (PMC11922610; doi:10.1155/jdr/8830658)
Supplement: Supporting Information 1 — Appendix Table S1: a comparison of the derivation and test set across the candidate variables. [file 8830658.f1.docx]

**Appendix Table 1: A comparison of the derivation and test set across the candidate variables**

|  | **Training set** | | **Test set** | |  |
| --- | --- | --- | --- | --- | --- |
|  | n = 457 | | n = 113 | | **p-value^a^** |
| **Age, mean (SD)** | 51.8 | 17.2 | 52 | 16.5 | 0.58 |
|  |  |  |  |  |  |
| **Race/Ethnicity, n (%)** |  |  |  |  | <0.01 |
| Non-Hispanic, White | 81 | 17.7 | 12 | 10.6 |  |
| Non Hispanic, Black or African American | 196 | 42.9 | 57 | 50.4 |  |
| Non Hispanic, Asian | 30 | 6.6 | 0 | 0.0 |  |
| Hispanic, Latino/a, or Spanish origin | 124 | 27.1 | 42 | 37.2 |  |
| Other/Unknown | 26 | 5.7 | 2 | 1.8 |  |
|  |  |  |  |  |  |
| **Sex, n (%)** |  |  |  |  | 0.95 |
| Female | 228 | 49.9 | 56 | 49.6 |  |
| Male | 229 | 50.1 | 57 | 50.4 |  |
|  |  |  |  |  |  |
| **Insurance type, n (%)** |  |  |  |  | 0.14 |
| Commercial / Private | 136 | 29.8 | 22 | 19.5 |  |
| Medicaid | 164 | 35.9 | 49 | 43.4 |  |
| Medicare | 116 | 25.4 | 29 | 25.7 |  |
| Other/unknown/uninsured | 41 | 9.0 | 13 | 11.5 |  |
|  |  |  |  |  |  |
| **Past medical history, n (%)** |  |  |  |  |  |
| Hypertension | 174 | 38.1 | 48 | 42.5 | 0.39 |
| Obesity | 191 | 41.8 | 47 | 41.6 | 0.97 |
| Hyperlipidemia | 49 | 10.7 | 15 | 13.3 | 0.44 |
| Cardiovascular Disease | 66 | 14.4 | 15 | 13.3 | 0.75 |
| Arthritis | 29 | 6.3 | 11 | 9.7 | 0.21 |
| COPD | 29 | 6.3 | 7 | 6.2 | 0.96 |
| Cancer | 39 | 8.5 | 13 | 11.5 | 0.33 |
| Substance Misuse | 41 | 9.0 | 12 | 10.6 | 0.59 |
| Heart Failure | 21 | 4.6 | 7 | 6.2 | 0.48 |
| Atrial Fibrillation | 21 | 4.6 | 7 | 6.2 | 0.48 |
|  |  |  |  |  |  |
| **Chief complaint included, n (%)** |  |  |  |  |  |
| Back pain | 15 | 3.3 | 2 | 1.8 | 0.55 |
| Fever/chills | 16 | 3.5 | 6 | 5.3 | 0.41 |
| Nausea/vomiting | 37 | 8.1 | 8 | 7.1 | 0.72 |
| ^a^Fischer exact test was used for where cell size smaller than 5 | | | | | |
