# Supplementary material for: Diabetes Screening in the Emergency Department: Development of a Predictive Model for Elevated Hemoglobin A1c
Source: J Diabetes Res. 2025 Mar 12;2025:8830658. doi: 10.1155/jdr/8830658 (PMC11922610; doi:10.1155/jdr/8830658)
Supplement: Supporting Information 2 — Appendix Table S2: frequency of variables selected across 40 models (excluding models with all variables). [file 8830658.f2.docx]

**Appendix Table 2: Frequency of variables selected across 40 models (Excluding models with all variables)**

| **Parameter** | **Frequency** | **Percentage** |
| --- | --- | --- |
| **Obesity** | 39 | 98% |
| **Substance misuse** | 39 | 98% |
| **Age** | 39 | 98% |
| **Hyperlipidemia** | 36 | 90% |
| **Insurance** | 32 | 80% |
| **Chief complaint: back pain** | 31 | 78% |
| **Chronic obstructive pulmonary disease** | 26 | 65% |
| **Chief complaint: fever chills** | 25 | 63% |
| **Race / ethnicity** | 22 | 55% |
| **Arthritis** | 9 | 23% |
| **Cancer** | 7 | 18% |
| **Sex** | 2 | 5% |
| **Chief complaint: nausea / vomiting** | 1 | 3% |
