# Supplementary figures and images for: Diabetes Screening in the Emergency Department: Development of a Predictive Model for Elevated Hemoglobin A1c
Source: J Diabetes Res. 2025 Mar 12;2025:8830658. doi: 10.1155/jdr/8830658 (PMC11922610; doi:10.1155/jdr/8830658)

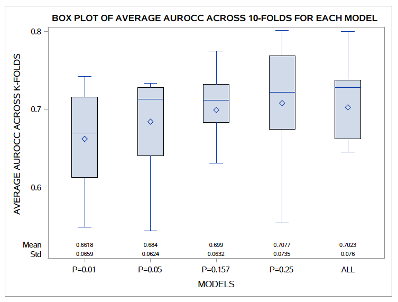

Supplement: Supporting Information 3 — Appendix Figure S1: box plot of the average C-statistic across the 10-folds for each model with varying p values. [file 8830658.f3.png]

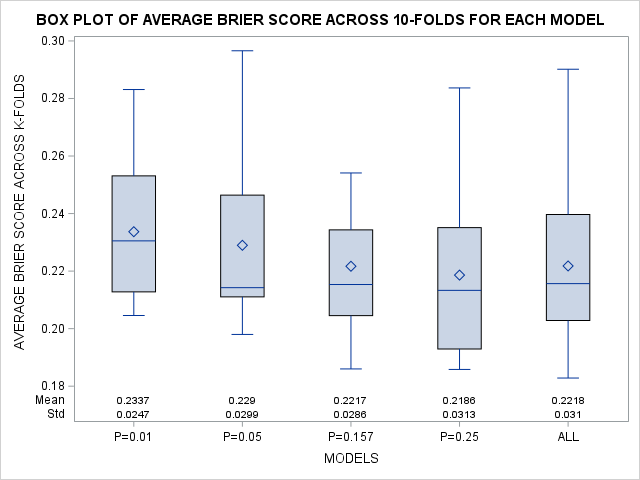

Supplement: Supporting Information 4 — Appendix Figure S2: box plot for the average Brier score across 10-folds for each model with varying p values. [file 8830658.f4.png]

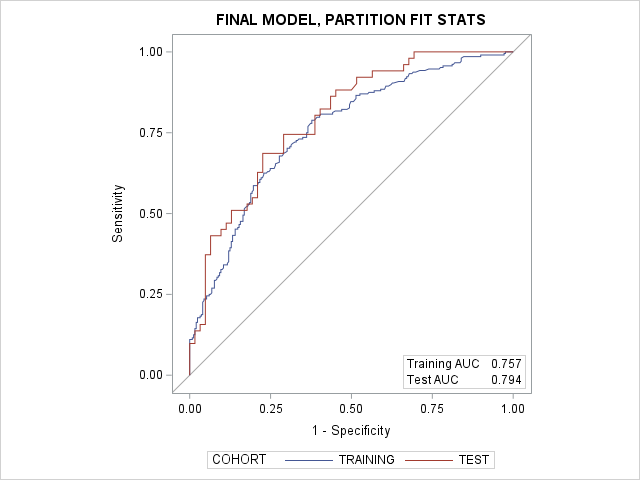

Supplement: Supporting Information 5 — Appendix Figure S3: the receiver operating characteristic curve (AUC) for the final model on the derivation and test cohort. [file 8830658.f5.png]
